# Trends in hospital admissions and prescribing due to chronic obstructive pulmonary disease and asthma in England and Wales between 1999 and 2020: an ecological study

**DOI:** 10.1186/s12890-023-02342-6

**Published:** 2023-02-02

**Authors:** Hassan Alwafi, Abdallah Y. Naser, Deema Sami Ashoor, Abdulelah M. Aldhahir, Jaber S. Alqahtani, Faisal Minshawi, Emad Salawati, Mohammed Samannodi, Mohammad Saleh Dairi, Aisha Khaled Alansari, Rakan Ekram

**Affiliations:** 1 Faculty of Medicine, Umm Al-Qura University, Mecca, Saudi Arabia; 2 Al-Noor Specialist Hospital, Mecca, Saudi Arabia; 3 Department of Applied Pharmaceutical Sciences and Clinical Pharmacy, Faculty of Pharmacy, Isra University, Amman, Jordan; 4 Respiratory Therapy Department, Faculty of Applied Medical Sciences, Jazan University, Jazan, Saudi Arabia; 5 Department of Respiratory Care, Prince Sultan Military College of Health Sciences, Dammam, Saudi Arabia; 6 Department of Laboratory Medicine, Faculty of Applied Medical Sciences, Umm Al-Qura University, Mecca, Saudi Arabia; 7 Department of Family Medicine, Faculty of Medicine, King Abdulaziz University, Jeddah, Saudi Arabia; 8 Maternity and Children Hospital, Ministry of Health, Mecca, Saudi Arabia; 9 School of Public Health and Health Informatics, Umm Al Qura University, Mecca, Saudi Arabia

**Keywords:** COPD, Asthma, Admissions, England, Wales, Hospitalization

## Abstract

**Objective:**

To investigate the trends in hospital admissions and medication prescriptions related to asthma and chronic obstructive pulmonary disease (COPD) in England and Wales.

**Methods:**

An ecological study was conducted between April 1999 and April 2020 using data extracted from the hospital episode statistics database in England and the patient episode database for Wales. The Office of National Statistics mid-year population estimates for 1999 through 2020 were collected, and medication prescription data for 2004–2020 were extracted from the prescription cost analysis database.

**Results:**

The total annual number of COPD and asthma hospital admissions for various causes increased by 82.2%, from 210,525 in 1999 to 383,652 in 2020, representing a 59.1% increase in hospital admission rate (from 403.77 in 1999 to 642.42 per 100,000 persons in 2020, *p* < 0.05). Chronic obstructive pulmonary disease with acute lower respiratory infection accounted for 38.7% of hospital admissions. Around 34.7% of all hospital admissions involved patients aged 75 and older. Around 53.8% of all COPD and asthma hospital admissions were attributable to females. The annual number of prescriptions dispensed for COPD and asthma medications increased by 42.2%.

**Conclusions:**

Throughout the study period, hospital admissions due to chronic obstructive pulmonary disease and asthma, as well as medication prescriptions, increased dramatically among all age groups. Hospitalization rates were higher for women. Further observational and epidemiological research is required to identify the factors contributing to increased hospitalization rates.

## Introduction

Globally, chronic respiratory diseases are among the top reasons for morbidity and mortality. Chronic obstructive pulmonary disease (COPD) and asthma are the most common chronic respiratory diseases [1]. Worldwide in 2019, the total number of people with COPD with age 30–79 is estimated to be 391.9 million (95% CI 312.6–487.9) utilizing the Global Initiative on Obstructive Lung Disease (GOLD) case definition [GOLD-COPD: FEV1/FVC < 0.7], and 292.0 million (95% CI 219.8–385.6) utilizing the lower limit of normal (LLN) definition [LLN-COPD: FEV1/FVC < LLN] [2]. In 2019, COPD caused 3.23 million deaths worldwide, standing as the third leading cause of death globally [3]. The estimated global number of asthma patients was about 262 million in 2019, and 461,000 deaths occurred due to asthma during the same year [4].

It is estimated that the number of people with COPD is more than 3 million in the United Kingdom (UK) [5]. The British Lung Foundation reports that 8 million individuals have been diagnosed with asthma accounting for more than 12% of the UK population [6]. In 2020/2021 in England, the national prevalence of asthma was 6.38%; as 3,629,071 patients (aged 6 years or older) were diagnosed with asthma on GPs’ registers, while the national prevalence of COPD was 1.93%; as 1,170,437 patients were diagnosed with COPD [7].

Over 128,000 people with a particular COPD exacerbation code in the UK were hospitalized, according to the National Health Services (NHS) Digital statistics in 2016/2017 [8]. However, 97% of those individuals were admitted to the emergency unit on average 3 days of their hospital length of stay [8]. In the UK, there are 5.4 million individuals who use asthma treatment, and 185 individuals are hospitalized with an asthma attack every day [9].

Lung disease is the fourth most costly condition in the UK, with an annual cost of £11 billion. Asthma and COPD are among the marked costly diseases, as asthma and COPD account for the cost of £3 billion and £1.9 billion annually, respectively [10]. Most COPD treatment costs are due to inpatient care, especially for acute COPD exacerbations at the hospital [11]. During the past 2 decades, the rate of hospital admission for chronic lower respiratory diseases (including asthma and COPD) was increased by 55.1% in England and Wales [12]. Moreover, annual healthcare costs and mortality costs for COPD are projected to increase to £2.32 billion by 2030 in England [13]. Accordingly, this research aimed to study the trend of admissions related to asthma and COPD and its associated medication prescriptions in England and Wales.

## Methods

### Study sources and the population

This was a cross-sectional ecological study using publicly available data extracted from the Hospital Episode Statistics (HES) database in England [14] and the Patient Episode Database for Wales (PEDW) for the period between April 1999 and April 2020 [15]. The HES and PEDW databases contain hospital admission data for patients with COPD and patients with asthma from all age groups which are subdivided into four categories; below 15 years, 15–59 years, 60–74 years, and 75 years and above. We identified COPD and asthma-related hospital admissions using the Tenth Revision of the International Statistical Classification of Diseases and Related Health Problems (ICD-10) 5th Edition (used by National Health Service (NHS) to classify diseases and other health conditions) (J44–J45) [16]. HES database records all admissions, A and E attendances and outpatient appointments at the National Health Service (NHS) hospitals in England. PEDW records all episodes of inpatient and day case activity in the National Health Service (NHS) Wales hospitals, which includes planned and emergency admissions, minor and major operations, and hospital stays for giving birth. Hospital activity for Welsh residents treated in other UK nations (primarily England) is also included. Data for hospital admissions in England and Wales are available from the years 1999/2000 onwards. Available data include patient demographics, clinical diagnoses, procedures, and duration of stay. HES and PEDW data are checked regularly to ensure their validity and accuracy [14, 17]. To calculate the yearly hospital admission rate for COPD and asthma, we collected mid-year population data for the period between 1999 and 2020 from the Office for National Statistics (ONS) [18].

COPD and asthma-related medication prescription data in England and Wales were extracted from the Prescription Cost Analysis database for the available period of 2004–2020 [19–21]. Data reported at the PCA database represent prescriptions dispensed and submitted to NHS Prescription Services. The British National Formulary (BNF) therapeutic classification system is used to report the PCA database data medication categories [22]. Prescription data became available in England from 2004 and in Wales from 2000. COPD and asthma medications were identified using the respiratory system chapter in the BNF (Chapter 3).

### Statistical analysis

Hospital admission rates with 95% confidence intervals (CIs) were calculated using the finished consultant episodes of COPD and asthma-related admission divided by the mid-year population. COPD and asthma medication prescription rates were calculated using the number of COPD and asthma medication prescriptions divided by the total mid-year population during the same year. We used the chi-squared to assess the difference between the admission rates in 1999 and 2020 and the difference between COPD and asthma medication prescription rates in 2004 and 2020. The trend in COPD and asthma-related hospital admission and COPD and asthma-related medication prescription were assessed using a Poisson model. All analyses were conducted using SPSS version 25 (IBM Corp, Armonk, NY, USA).

## Results

The total annual number for COPD and asthma hospital admissions per ICD codes for various causes increased by 82.2% from 210,525 in 1999 to 383,652 in 2020, representing an increase in hospital admission rate of 59.1% [from 403.77 (95% CI 402.05–405.49) in 1999 to 642.42 (95% CI 640.39–644.45) in 2020 per 100,000 persons, trend test, *p* < 0.05]. The most common COPD and asthma hospital admissions causes were chronic obstructive pulmonary disease with acute lower respiratory infection, asthma, and chronic obstructive pulmonary disease with acute exacerbation, unspecified which accounted for 38.7%, 30.0%, and 25.5%, respectively (Table 1).

**Table 1:** Percentage of COPD and asthma from total number of admissions and percentage change in admission rates

ICD code	Description	Percentage from total number of admissions (%)	Rate of admissions in 1999 per 100,000 persons (95% CI)	Rate of admissions in 2020 per 100,000 persons (95% CI)	Percentage change from 1999 to 2020 (%)
J44.0	“Chronic obstructive pulmonary disease with acute lower respiratory infection”	38.7	105.21 (104.33–106.09)	314.07 (312.65–315.48)	198.5
J44.1	“Chronic obstructive pulmonary disease with acute exacerbation, unspecified”	25.5	116.85 (115.93–117.78)	111.28 (110.43–112.12)	− 4.8
J44.8	“Other specified chronic obstructive pulmonary disease”	0.4	2.89 (2.74–3.04)	1.67 (1.56–1.77)	− 42.4
J44.9	“Chronic obstructive pulmonary disease, unspecified”	5.3	45.23 (44.65–45.81)	20.17 (19.81–20.53)	− 55.4
J45	Asthma (Predominantly allergic asthma, nonallergic asthma, mixed asthma, and asthma, unspecified)	30.0	133.59 (132.60–134.58)	195.24 (194.12–196.36)	46.1

*ICD* international statistical classification of diseases system

**Fig. 1:**
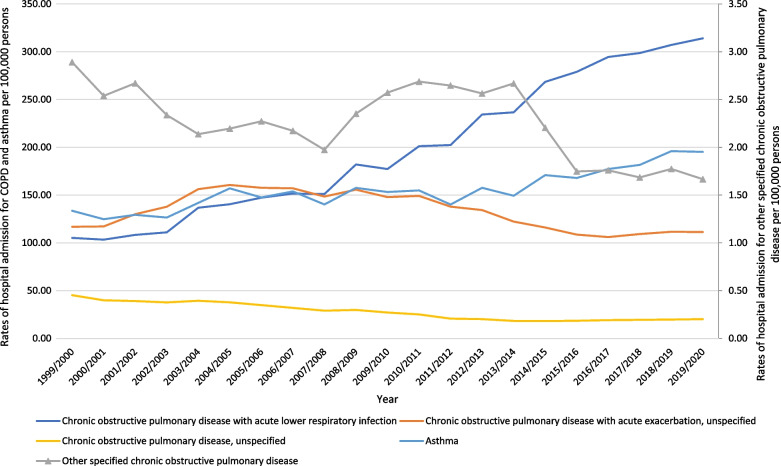
Rates of hospital admission for COPD and asthma in England and Wales stratified by type between 1999 and 2020

**Fig. 2:**
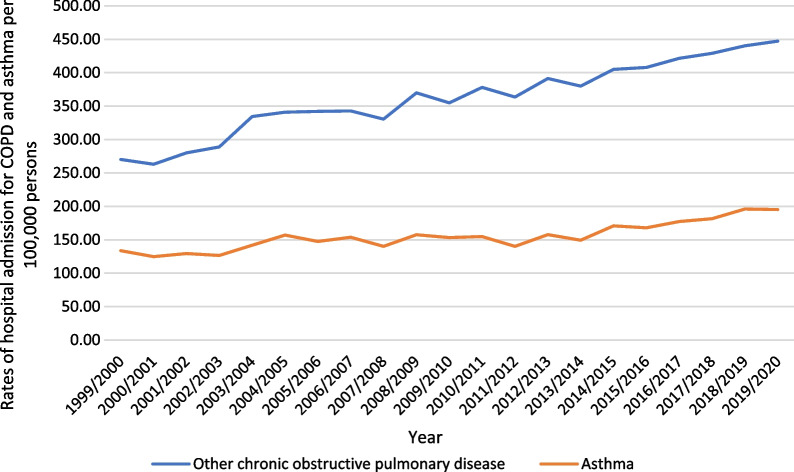
Rates of hospital admission for COPD and asthma in England and Wales between 1999 and 2020

Over the past 21 years, a considerable increase in COPD hospital admissions rate was seen in chronic obstructive pulmonary disease with acute lower respiratory infection by 1.99-fold. Besides, the hospital admissions rate for asthma increased by 46.1%. However, COPD hospital admissions rate for chronic obstructive pulmonary disease, unspecified, other specified chronic obstructive pulmonary disease, and chronic obstructive pulmonary disease with acute exacerbation, unspecified were decreased by: 55.4%, 42.4%, and 4.8%, respectively (Fig. 1). The overall rate of admissions related to COPD increased by 65.5%, Fig. 2.

**Fig. 3:**
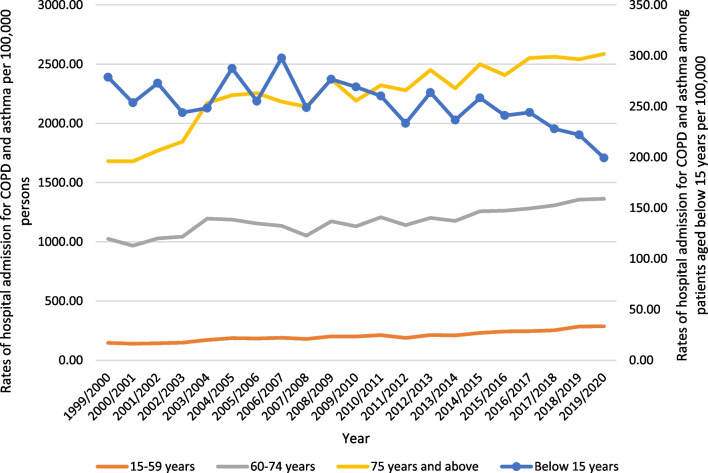
Rates of hospital admission for COPD and asthma in England and Wales stratified by age group

Regarding age group diversity for COPD and asthma hospital admission, the age group 75 years and above accounted for 34.7% of the total number of COPD and asthma hospital admissions, followed by the age group 60–74 years with 33.0%, the age group 15–59 years with 23.5%, and then the age group below 15 years with 8.8%. Rates of hospital admission for COPD and asthma among patients aged below 15 years decreased by 28.5% [from 278.84 (95% CI 275.56–282.13) in 1999 to 199.34 (95% CI 196.67–202.01) in 2020 per 100,000 persons]. Rates of hospital admission for COPD and asthma among patients aged 15–59 years increased by 96.3% [from 146.35 (95% CI 145.01–147.69) in 1999 to 287.26 (95% CI 285.48–289.05) in 2020 per 100,000 persons]. Rates of hospital admission for COPD and asthma among patients aged 60–74 years increased by 33.0% [from 1025.32 (95% CI 1017.82–1032.82) in 1999 to 1363.34 (95% CI 1355.91–1370.78) in 2020 per 100,000 persons]. Rates of hospital admission for COPD and asthma among patients aged 75 years and above increased by 53.8% [from 1681.08 (95% CI 1668.35–1693.82) in 1999 to 2586.24 (95% CI 2572.56–2599.92) in 2020 per 100,000 persons] (Fig. 3).

**Fig. 4:**
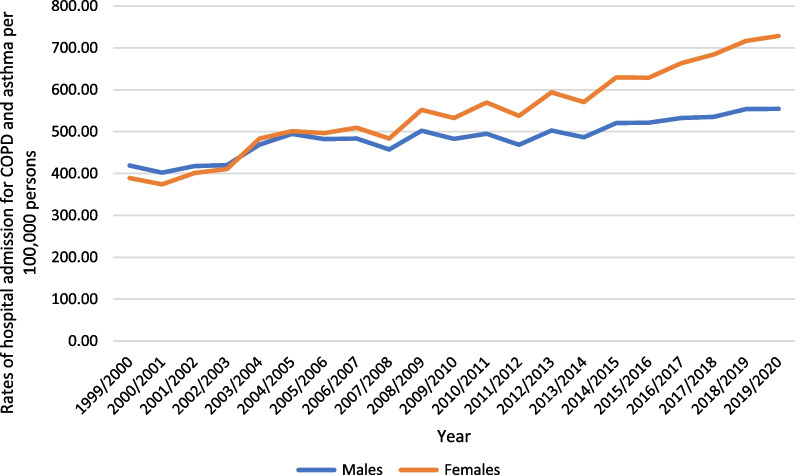
Rates of hospital admission for COPD and asthma in England and Wales stratified by gender

A total of 6,068,837 COPD and asthma hospital admission episodes were reported in England and Wales during the study duration. Females contributed to 53.8% of the total number of COPD and asthma hospital admission accounting for 3,264,503 hospital admission episodes by a mean of 155,452 per year. COPD and asthma hospital admission rate between females was increased by 87.2% [from 389.14 (95% CI 386.78–391.50) in 1999 to 728.40 (95% CI 725.37–731.44) in 2020 per 100,000 persons]. COPD and asthma hospital admission rate between males was increased by 32.3% [from 419.09 (95% CI 416.58–421.60) in 1999 to 554.48 (95% CI 551.80–557.16) in 2020 per 100,000 persons] (Fig. 4).

### COPD and asthma admission rate by gender

**Fig. 5:**
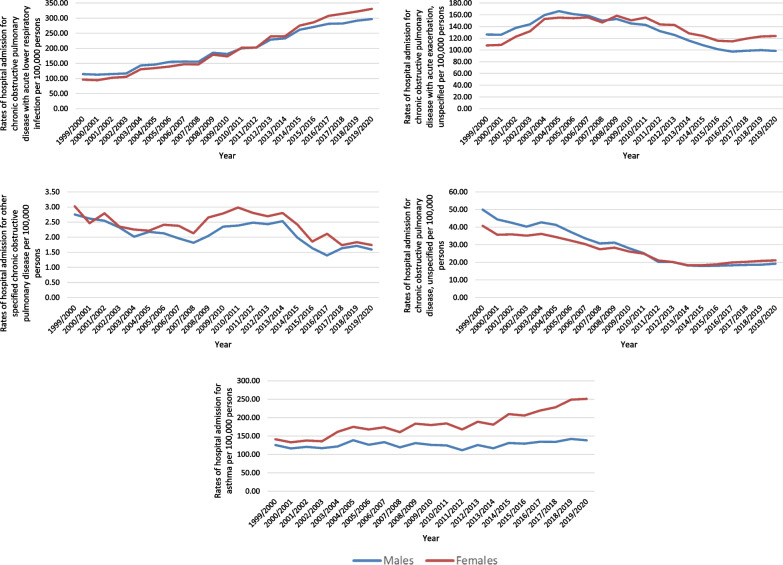
Hospital admission rates for COPD and asthma in England and Wales stratified by gender

COPD and asthma hospital admission rates were higher among females compared to males except for chronic obstructive pulmonary disease, unspecified, which were higher among males compared to females (Fig. 5).

### COPD and asthma admission rate by age group

**Fig. 6:**
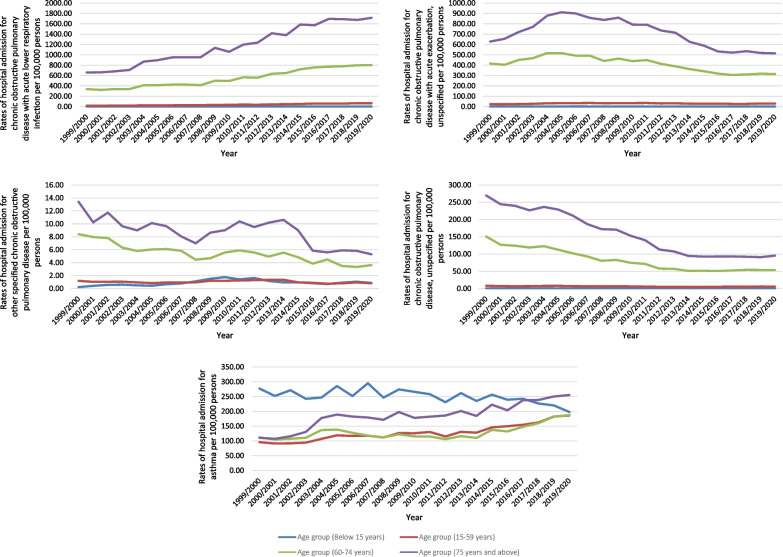
Hospital admission rates for COPD and asthma in England and Wales stratified by age group

COPD-related hospital admissions were seen to be directly related to age (more common among the age group 75 years and above). That includes the following: chronic obstructive pulmonary disease with acute lower respiratory infection, chronic obstructive pulmonary disease with acute exacerbation, unspecified, other specified chronic obstructive pulmonary disease, and Chronic obstructive pulmonary disease, unspecified. Still, asthma-related hospital admissions were more common among the age group: below 15 years, 75 years and above, 60–74 years, and 15–59 years, respectively (Fig. 6).

### COPD and asthma medication prescriptions

**Fig. 7:**
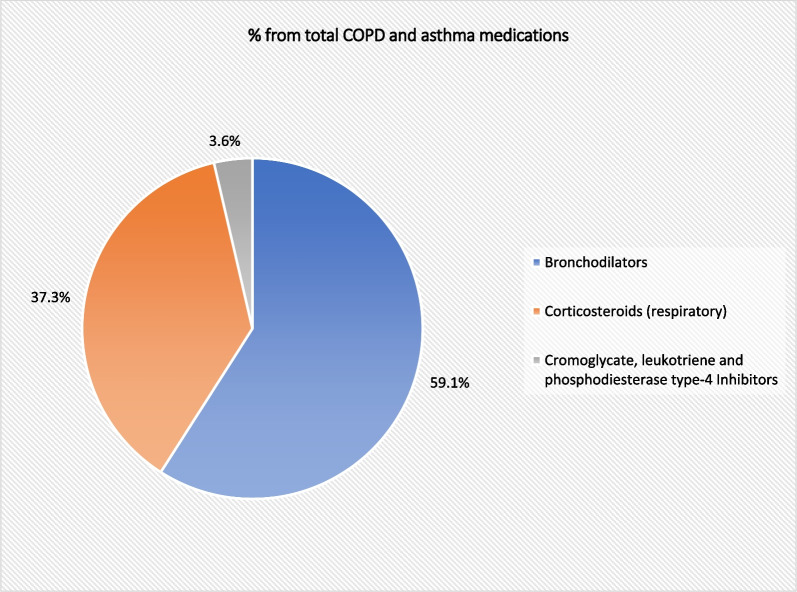
Percentage of each medication prescription from the total number of COPD and asthma medication prescriptions

The absolute number of COPD and asthma medications prescriptions dispensed annually in England and Wales increased by 42.2% [from 42,062,859 in 2004 to 59,819,658 in 2020]. COPD and asthma medications prescription rates increased by 27.2% [from 78,745.51 (95% CI 78,722.67–78,768.35) in 2004 to 100,167.34 (95% CI 100,143.26–100,191.42) in 2020 prescriptions per 100,000 persons, trend test, *p* < 0.05]. The most common COPD and asthma medication prescriptions were bronchodilators and corticosteroids (respiratory), which accounted for: 59.1% and 37.3%, respectively (Fig. 7).

**Fig. 8:**
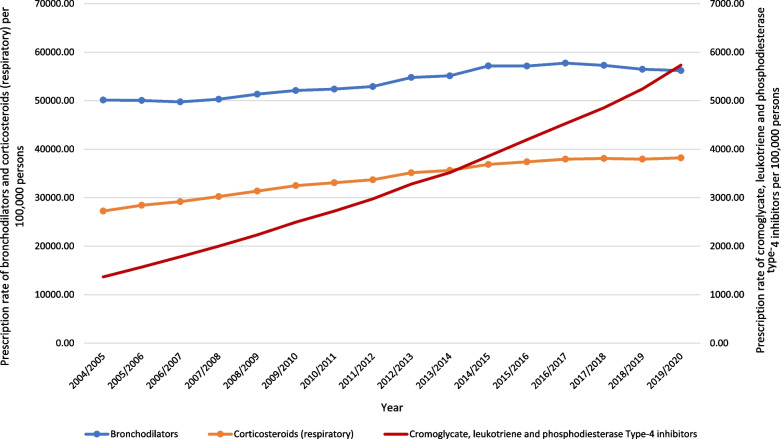
Prescription rates of COPD and asthma medications in England and Wales between 2004 and 2020

**Fig. 9:**
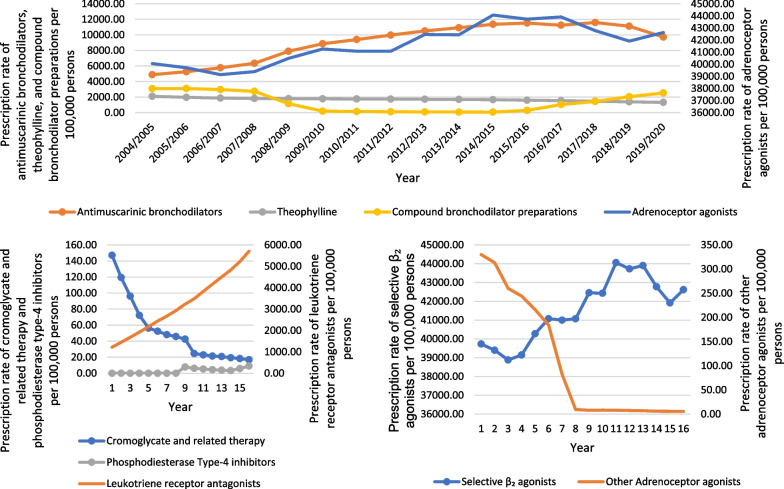
Prescription rates of COPD and asthma medications subtype in England and Wales between 2004 and 2020

During the past 16 years, a tremendous increase in the rate of COPD and asthma medication prescriptions was noticed in cromoglycate, leukotriene and phosphodiesterase type-4 inhibitors with 3.20-fold. Besides, the rate of COPD and asthma medication prescriptions for corticosteroids (respiratory) and bronchodilators was increased by 40.3% and 12.1%, respectively (Table 2, Figs. 8, and 9).

**Table 2:** Percentage change in COPD and asthma prescriptions rates from 2004 to 2020 with 95% CI

COPD and asthma medications	Prescriptions rate in 2004 prescriptions per 100,000 persons (95% CI)	Prescriptions rate in 2020 prescriptions per 100,000 persons (95% CI)	% change from 2004 to 2020 (%)	95% confidence interval
*Bronchodilators*				
Total	50,132.91 (50,119.50–50,146.31)	56,217.53 (56,204.95–56,230.12)	12.1	11.31–12.96
Adrenoceptor agonists				
Total	40,059.54 (40,046.40–40,072.68)	42,624.80 (42,612.25–42,637.34)	6.4	5.78–7.02
Selective β_2_ agonists	39,729.43 (39,716.31–39,742.55)	42,619.21 (42,606.67–42,631.75)	7.3	6.64–7.96
Other adrenoceptor agonists	330.11 (328.57–331.65)	5.59 (5.40–5.78)	− 98.3	− 97.97 to − 98.63
Antimuscarinic bronchodilators	4884.60 (4878.82–4890.38)	9733.11 (9725.60–9740.63)	99.3	99.09–99.51
Theophylline	2098.36 (2094.51–2102.20)	1322.37 (1319.47–1325.26)	− 37.0	− 35.78 to − 38.22
Compound bronchodilator preparations	3090.41 (3085.77–3095.05)	2537.26 (2533.27–2541.24)	− 17.9	16.93–18.87
*Inhaled corticosteroids*	27,246.10 (27,234.16–27,258.04)	38,216.42 (38,204.09–38,228.74)	40.3	39.06–41.54
*Cromoglycate, leukotriene and phosphodiesterase type-4 Inhibitors*				
Total	1366.51 (1363.39–1369.62)	5733.39 (5727.49–5739.29)	319.6	318.42–320.78
Cromoglycate and related therapy	147.11 (146.08–148.14)	16.93 (16.60–17.26)	− 88.5	87.69–89.31
Leukotriene receptor antagonists	1219.40 (1216.45–1222.34)	5707.33 (5701.45–5713.21)	368.0	366.78–369.22
Phosphodiesterase type-4 inhibitors	< 0.01 (0.00–0.01) *In 2011	9.13 (8.89–9.37)	516,325.1 *from 2011	516,323.73–516,326.27

## Discussion

More than 500,000 thousands cases of respiratory disease, including COPD and asthma, are diagnosed annually in the UK [23]. This study aimed to estimate the trend of hospital admission associated with COPD and asthma in the UK from 1999 to 2020; moreover, we describe the type of prescriptions for COPD and asthma. Our study showed that the admission rates of COPD and asthma in England and Wales increased by an average of 3.9% each year. According to previous studies, hospitalisation rates for asthma and COPD have increased in the UK during the past few years [12, 24]. A study conducted in England and Wales between 2002 and 2011 showed an increase in asthma-related admissions to adult intensive care units by an average of 4.7% per year across the 10 years [25].

This study closely observed the effect of age group and gender on COPD and asthma-related hospital admission. Regarding age group, the rate of hospital admission related to COPD and asthma increased in adult and elderly patients; however, hospital admission in childhood (< 15) decreased. Our previous study showed the trend of hospital admissions due to respiratory diseases, including influenza and pneumonia, and chronic lower respiratory were age-dependent [26]. One way to interpret this finding is that COPD and asthma are frequently underestimated due to the atypical presentation and associated comorbidities in adults [27]. Further, the prevalence of both asthma and COPD in the UK is increasing with aging, which indicate that additional cases that have not been identified are being found, or it may indicate that the risk factors such as smoking and air pollution are growing more prevalent [28, 29]. This is also true in other high-income countries, as well as low-income and middle-income countries, where the burden of these two disorders becomes more intense as people become older [30, 31].

Some comorbidities emerge independently of COPD, whereas others are accompanying to COPD [32]. Comorbidities such as cardiac disease, diabetes mellitus, and hypertension are commonly reported in patients with COPD [33]. On the other hand, asthma is usually accompanied by several comorbidities, which may influence its clinical manifestation and severity [34]. In England, the proportion of adults who reported having at least one chronic condition grew from 40% in 1993 to 43% in 2019 [35]. In addition, the rate of doctor-diagnosed diabetes has climbed from 2 to 7% from 1994 to 2019 [36]. Therefore, the age-dependent onset is an essential factor in helping the management of COPD and asthma. We believe that the awareness toward COPD and asthma is increasing over time and directly impacts the early diagnosis and management of these diseases. A previous cohort study showed that asthma-related outcomes depend on age at onset [37].

Likewise, our data found that female admissions were slightly higher than male admissions over 21 years, accounting for 53.8% of all COPD and asthma-related hospital admissions. It is well known that the global prevalence of asthma is higher in females than in males and that the prevalence of COPD is higher in males than in females [38, 39]. Besides, recent data also showed that the trend of COPD among females is rising. A previous study reported that the rates of admissions related to chronic lower respiratory diseases in England and Wales between 1999 and 2019 were higher among females than males [26]. In addition, another study showed that between 2002 and 2011 in England and Wales that 67% of acute asthma were females, and 33% were males [25]. This might be because men and women have different levels of sex hormones, have a different aetiology for airway hyperreflexia, or have different immunology [40, 41]. In addition, this could be explained due to the fact that females have more extended life expectancy, which ultimately will increase the accumulative number of patients susceptible for respiratory diseases complications and its associated admissions [42]. Besides, females contracting an increasing number of respiratory diseases worldwide [41]. Previous literature reported that more females died from COPD than males globally, which reflects the dynamic change in disease prevalence [43].

In terms of medicine prescriptions, our study found that as the rate of hospitalization increased, the medication used to treat COPD and asthma risen by 2.6% each year on average. A previous study found that the ratio of salbutamol to inhaled corticosteroids prescription increased gradually in England from 2013 to 2017 as the ratio rose from 0.65 to 0.69 [24]. It is remarkable to demonstrate that type of medication changed over time, and national and international guidelines have also changed. For instance, the use of a biological therapy such as leukotriene receptor antagonists increased 368% from 2004 to 2020. However, a study conducted in East London between 1991 and 1994 found that practices with higher prescribing ratios had lower hospital admission rates [44].

The increase in the prescribing of respiratory disease medications can be justified by the increase in the prevalence of both COPD and asthma. However, a justification of the increase in the admission rates of COPD and asthma despite the increase in the prescribing can be due to poor disease management related to medication non-adherence. Nonetheless, this highlights the need for personalized management for patients with COPD and asthma and enhancing patients’ awareness concerning the importance of medications adherence for a better control of the diseases. However, future studies including qualitative studies to investigate risk factors that may be linked to higher admission rates and/or poor adherence are needed.

The study has many limitations. First, it is challenging to individualize the results because ecological studies base their findings on population averages. Second, this was an ecological study on the population level not on individual level, which restricted our ability to retrieve data related to comorbidities, medications history and adherence, and laboratory history, that might also influence patient outcomes. Third, the outcomes of the study were identified using relevant Read code lists which may be influenced by the coding behaviour and therefore, underreporting or overestimation of the outcomes cannot be ruled out. Four, due to the aggregated nature of the data on the population level, we were unable to estimate the age-adjusted rate of admissions. However, the data we have used, have been validated for epidemiological research purposes, and has been used for the description of various diseases outcomes across the UK [45–47]. In addition, there is a possibility of systematic differences across regions in tracking the disease frequency and measuring exposures. Moreover, the lack of available data on factors including physical inactivity and detailed prevalence of specific diseases may have a role in the changes in admission rates directly or indirectly.

## Conclusion

This study utilizes a large dataset stratified by age and gender, providing a detailed description of the hospitalization profile of patients across England and Wales over 21 years and the availability of prescribing data over 16 years. We found that hospital admissions due to COPD and asthma, as well as medication prescriptions, increased dramatically among all age groups. Further observational and epidemiological research is required to identify the factors contributing to increased hospitalization rates.

## Data Availability

The datasets used and/or analysed during the current study are available from the corresponding author on reasonable request.

## Abbreviations

GOLD: Global initiative on obstructive lung disease
LLN: Lower limit of normal
UK: United Kingdom
COPD: Chronic obstructive pulmonary disease
HES: Hospital episode statistics
PEDW: Patient episode statistics for Wales
SPSS: Statistical package for social sciences
ICD: International classification of diseases
CI: Confidence interval
